# Application of seventeen two-locus models in genome-wide association studies by two-stage strategy

**DOI:** 10.1186/1753-6561-3-s7-s26

**Published:** 2009-12-15

**Authors:** Adan Niu, Zhaogong Zhang, Qiuying Sha

**Affiliations:** 1 Department of Mathematical Sciences, Michigan Technological University, Houghton, Michigan 49931, USA; 2 School of Computer Science and Technology, Heilongjiang University, Harbin 150080, PR China

## Abstract

The goal of this paper is to search for two-locus combinations that are jointly associated with rheumatoid arthritis using the data set of Genetic Analysis Workshop 16 Problem 1. We use a two-stage strategy to reduce the computational burden associated with performing an exhaustive two-locus search across the genome. In the first stage, the full set of 531,689 single-nucleotide polymorphisms was screened using univariate testing. In the second stage, all pairs made from the 500 single-nucleotide polymorphisms with the lowest *p*-values from the first stage were evaluated under each of 17 two-locus models. Our analyses identified a two-locus combination - rs6939589 and rs11634386 - that proved to be significantly associated with rheumatoid arthritis under a Rec × Rec model (*p*-value = 0.045 after adjusting for multiple tests and multiple models).

## Background

Although the primary interest of genome-wide association studies (GWAS) is to identify single-nucleotide polymorphisms (SNPs) with detectable marginal effects, it is also of interest to identify SNPs that have an interaction effect. Testing all possible pairs of loci to identify interactions creates many practical difficulties. In this article, we use a two-stage strategy to search for two-locus joint effects, which effectively reduces the computation time. In the first stage, we only selected loci that met an initial threshold. In the second stage, any locus that met the first-stage threshold was tested under each of 17 two-locus joint effect models. By applying this two-stage analysis to Genetic Analysis Workshop 16 (GAW16) Problem 1, we successfully identified two SNPs that are jointly associated with rheumatoid arthritis (RA) and could not be identified by single-locus analysis.

## Methods

### Two-locus analysis based on 17 two-locus models

**Figure 1:**
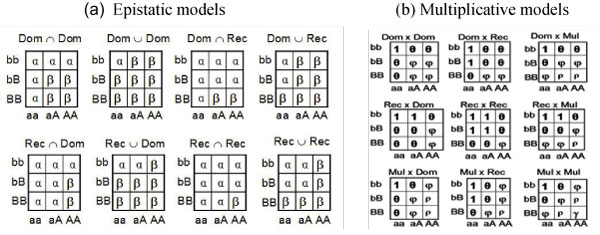
**Seventeen two-locus models**. A and B are the high-risk alleles in the two markers. For the epistatic models, *α *and *β *are the penetrances. For the multiplicative models, the symbol in each cell denotes the relative risk of this cell. *φ *= *θ*^2^, *ρ *= *θ*^3^, and *γ *= *θ*^4^.

In this article, we propose 17 two-locus models, which include 8 epistatic models and 9 multiplicative models (Figure 1).

The 17 two-locus models can be described by the following log linear model log *P*(*Disease*|*g*) = *β*_0 _+ *β*_1_*X*, where *g *is the two-locus genotype; for the eight epistatic models in which the nine two-locus genotypes can be divided into high-risk group and low-risk group, *X *= 1 if the genotype *g *belongs to the high-risk group and *X *= 0 otherwise; for the nine multiplicative models, *X *= *x*_1 _+ *x*_2_, where *x*_1 _is the dominant, recessive, or additive coding of the genotype at the first SNP for a dominant, recessive, or multiplicative model, respectively, and *x*_2 _is similarly defined for the second SNP. For the *i*^th ^individual, let *y*_*i *_denote the trait value (1 for diseased individual and 0 for normal individual) and *X*_*i *_denote the numerical code of the genotype (*X *in the log linear model). The score test statistic is given by

where *N *is the sample size,  is the average of *X*_1_, ..., *X*_*N*_, and  is the mean of *y*_1_, ..., *y*_*N*_. Under null hypothesis of no association, *T*_*score *_asymptotically follows a chi-square distribution with one degree of freedom (df). Though we use a unified test statistic *T*_*score *_for all of the models, the 17 models correspond to 17 different tests. Different models correspond to different genotype coding, and thus different values of *X*_*i *_in *T*_*score*_. For example, under Rec ∩ Rec epistatic model, *X *= 1 if the two-locus genotype is AABB; *X *= 0 otherwise; under Rec× Rec multiplicative model, *X *= *x*_1 _+ *x*_2_, where *x*_1 _= 1 if the genotype at the first SNP is AA and *x*_1 _= 0 otherwise; *x*_2 _is similarly defined for the second SNP.

The method to search for significant two-locus combinations for each of the 17 models has the following two steps. In the first step, three one-df chi-square tests (corresponding to dominant, recessive, and additive models) are used to test for association at each SNP. For a SNP, let *P *denote the smallest *p*-value of the three tests. We select *M *SNPs with the smallest *P *(*M *= 500 was used in this study). In the second step, under each of the 17 two-locus models, we apply a two-locus association test *T*_*score *_to each of the *L *two-locus combinations among the *M *selected SNPs, where *L *= *M*(*M *- 1)/2. In this step, we get a *p*-value (called raw *p*-value) for each of the *L *two-locus combinations and each of the 17 two-locus models.

Let *p*_*ij *_denote the raw *p*-value of the *l*^th^two-locus combination under the *j*^th ^two-locus model. A permutation procedure is then used to adjust for multiple tests and multiple models. For each permutation, we randomly shuffle the case and control status. Based on the permuted data, we redo the single-marker test for each SNP, select *M *SNPs with the smallest *p*-values, and then test each of the two-locus combinations among the *M *selected SNPs under each of the 17 models. For the *k*^th ^permutation, let  denote the *p*-value of the two-locus test for the *l*^th ^two-locus combinations under the *j*^th ^model and let . Suppose that we perform the permutation *K *times (we use *K *= 1000 in this study). Then, the overall *p*-value of the two-locus test for the *l*^th ^two-locus combination under the *j*^th ^model is given by . In this way, we can adjust for multiple testing for *L *two-locus tests and 17 models and we also can adjust for the fact that the *M *SNPs to do two-locus analysis were chosen as the "top" signals from a much larger set.

#### Control for population stratification

**Figure 2:**
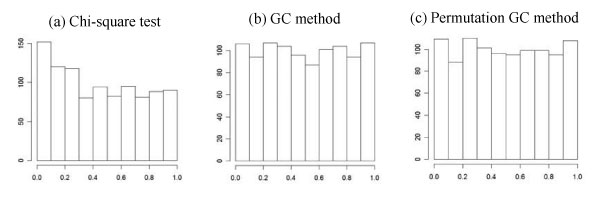
**Histograms of the *p*-values of the three tests**.

The two-locus analysis discussed in the last section is valid under the assumption that all sampled individuals come from a homogeneous population. Thus, we first check this assumption in the data set of GAW16 Problem 1. For this purpose, we randomly choose 1000 SNPs and perform association test at each of the 1000 SNPs using the one-df chi-square test. The histogram of the *p*-values of the 1000 tests is given in Figure 2a. If all sampled individuals come from a homogeneous population, the 1000 *p*-values should follow a uniform distribution. Figure 2a shows that the data set of GAW16 Problem 1 has the problem of population stratification. To control for population stratification, we propose to use the following two methods:

1. Genome Control (GC) method: the GC method [1,2] rescales the chi-square test statistic. Suppose that we have *N *one-df chi-square tests (single-marker test or two-locus test) with test statistics *T*_1_, *T*_2_, ..., *T*_*N*_. The GC method rescales *T*_*i *_as  (*i *= 1, 2, ..., *N*), where *b *= *median *{*T*_1_, *T*_2_, ..., *T*_*N*_}/0.465 and considers *S*_*i *_following a chi-square distribution with one df.

2. Permutation GC method: Suppose that we have *N* one-df chi-square tests and rescale the test statistics as *S*_1_, *S*_2_, ..., *S*_*N *_by the GC method. Then, we carry out permutation *K *times. For each permutation, we perform *N *chi-square tests based on the permuted data and rescale the test statistics by GC method. For the *k*^th ^permutation, let  denote the chi-square test statistics. We rescale them as  by GC method. Then, the *p*-value of the *i*^th ^test is given by .

To test whether the two methods can control for population stratification, we randomly chose 1000 SNPs from the data set of GAW16 Problem 1. The GC and permutation GC methods were used to test association for the 1000 SNPs. The histograms of the *p*-values from the two methods are shown in Figure 2, b and c. These two panels show that the distributions of the *p*-values are very similar to a uniform distribution, which indicates that both the GC and permutation GC methods can control for population stratification, at least for the data set of GAW16 Problem 1.

## Results

The GAW16 Problem 1 data set contains genotypes at 531,690 SNPs on chromosomes 1-22 (868 cases and 1194 controls). We first performed single-marker analysis. Three one-df chi-square tests (corresponding to dominant, recessive, and additive models) rescaled by GC method were applied to each SNP. The smallest *p*-value of the three tests at each SNP was adjusted by Bonferroni correction for the three models and all the SNPs. From the single-marker analysis, we found one SNP (rs2476601) on chromosome 1 and 183 SNPs on chromosome 6 with adjusted *p*-values less than 0.05 after Bonferroni correction. The 183 SNPs that we found on chromosome 6 are in a region (29930240 to 33149012 bp) in high linkage disequilibrium with HLA-DRB1, a factor known to have a strong association with RA [3-6]. The SNP (rs2476601) on chromosome 1 is in the hematopoietic-specific protein tyrosine phosphatase gene, *PTPN22*, which has been identified to be associated with RA [6,7].

We used the permutation GC method with 1000 permutations to carry out the two-locus analysis. In the analysis, we excluded SNPs within 10 Mbp of any of the SNPs surviving Bonferroni correction from the single-marker analysis. Then, we performed the two-stage, two-locus analysis among the remaining SNPs. For each pair of SNPs, individuals with missing genotypes at either locus were not included in that analysis. After adjusting for multiple tests and multiple SNPs, we found one two-locus combination - rs6939589 on chromosome 6 and rs11634386 on chromosome 15 - significantly associated with RA. The two-locus combination followed the Rec× Rec multiplicative model with a *p*-value of 0.045.

## Discussion

Although there is growing appreciation that searching for epistatic interactions in humans may be a fruitful endeavor, there are still a number of practical difficulties associated with testing all possible pair-wise comparisons in the case of GWAS, such as data storage requirements, computation time, and multiple testing. In this report we used a computationally efficient two-stage analysis to search for joint effects of genes for GAW16 Problem 1. Applying this strategy to GAW16 Problem 1, we have successfully identified two loci that are significantly associated with RA. These two loci would not have been identified as significant by single-marker analysis after correction for multiple tests.

Computational efficiency has imposed limits on our two-stage strategy. Because our approach only searches two-locus interaction among the top *M *SNPs according to the marginal effects, the loci with strong interaction effects but weak marginal effects may not be detected. Furthermore, our approach only considers 17 interaction patterns (corresponding to the 17 two-locus models). The proposed two-stage strategy may have low power to detect interactions whose patterns depart from the 17 two-locus models.

## Conclusion

Our two-locus analysis showed that a two-locus combination, rs6939589 and rs11634386, is significantly associated with RA.

## List of abbreviations used

GAW16: Genetic Analysis Workshop 16; GC: Genome control; GWAS: Genome-wide association study; RA: Rheumatoid arthritis; SNP: Single-nucleotide polymorphism

## Competing interests

The authors declare that they have no competing interests.

## Authors' contributions

AN performed the statistical analysis and wrote the first draft of the manuscript. ZZ participated in the study design and helped to draft the manuscript. QS contributed to the design of the study and to the manuscript preparation. All authors read and approved the final manuscript.
